# Assessment of the Radiation Attenuation Properties of Several Lead Free Composites by Monte Carlo Simulation

**Published:** 2015-06-01

**Authors:** M. Kazempour, M. Saeedimoghadam, F. Shekoohi Shooli, N. Shokrpour

**Affiliations:** 1 Student Research Committee, Shiraz University of Medical Sciences, Shiraz, Iran;; 2 Department of Radiology, School of paramedical sciences, Shiraz University of Medical Sciences, Shiraz, Iran;; 3 Ionizing and Non-Ionizing Radiation protection research center, School of Paramedical Sciences, Shiraz University of Medical Sciences, Shiraz, Iran;; 4 Shahid Beheshti University of Medical Science, Tehran, Iran; 5 English department, faculty of Paramedical Sciences, Shiraz University of Medical Sciences, Shiraz, Iran

**Keywords:** Lead free sheilds, MCNP4C, Diagnostic radiology energy range, Radiation protection garments, Narrow beam and broad beam geometry

## Abstract

**Background:**

In diagnostic radiology lead apron, are usually used to protect patients and radiology staff against ionizing radiation. Lead apron is a desirable shield due to high absorption and effective attenuation of x-ray photons in the diagnostic radiology range.

**Objective:**

Although lead aprons have good radiation protection properties, in recent years, researchers have been looking for alternative materials to be used instead of lead apron because of some problems derived from lead-content of aprons. Because of its lead-content, these radiation protection garments are so heavy and uncomfortable for the staff to wear, particularly in long-time uses. In addition, lead is a toxic element and its disposal is associated with environmental and human-health hazards.

**Method:**

In this study, several new combinations of lead free materials ((W-Si), (W-Sn-Ba-EPVC ), (W-Sn-Cd-EPVC)) have been investigated in the energy range of diagnostic radiology in two geometries: narrow and broad beam. Geometries of the radiation attenuation characteristics of these materials was assessed in 40, 60, 90 and 120 kVp and the results compared with those of some lead-containing materials ((Pb-Si), (Pb-EPVC)).

**Results:**

Lead shields still provide better protection in low energies (below 40 kVp). Combination of W-Sn-Cd-EPVC has shown the best radiation attenuation features in 60 and 90 kVp and the composition of (W-Sn-Ba-EPVC) represents the best attenuation in 120 kVp, even better than previously mentioned lead- containing composites.

**Conclusion:**

Lead free shields are completely effective for protection against X-ray energies in the range of 60 to 120 kVp.

## Introduction

Lead-based garments are generally useful in diagnostic radiology departments because of their effective attenuation of photons. For years, lead shields have been used in radiology, nuclear medicine, interventional angiography procedures, etc. to protect patients and radiation staff against ionizing radiation[1]. Conventionally aprons made of lead have been used in diagnostic radiology and interventional trials because of their extraordinary efficiency in reducing radiation doses in patients and operators, respectively. Without these shields, direct and secondary exposure to ionizing radiation might lead to biological damages in healthy tissues.

Although lead shields are so beneficial to mitigate radiation doses reaching patient and radiation staff, questions have been raised about the safety of prolonged use of them. Because of the density of lead, these shields are so heavy, so its carrying is a burdensome task especially in long procedures, for example in interventional angiography, as Moore et al. demonstrated the relationship between the use of lead aprons and development of back pain[2]. Moreover, since lead is a toxic element, its long use may endanger the user’s health[3-4]. Recently, researchers have shown an increased interest in looking for alternative non-toxic materials with less weight and possibly same attenuation to use instead of lead to overcome its mass and toxicity problems[5-10]. For example, in 2007, J. P. McCaffrey et al. showed that some lead free materials may offer better reduction in radiation transition than lead-based materials per unit weight, especially in the keV region above the K-absorption edges of lead free element. They claimed at mean x-ray photon energies less than about 45 keV, some lead free based materials may offer somewhat better attenuation per unit mass than even pure lead[11]. Another study indicated that significantly enhanced radiation attenuation per unit mass can be obtained using bilayers to produce radiation protection garments[12]. Bilayers used in radiation protection, consist of two different layers of radiation attenuating materials used as a unique layer, with lower-Z element placed near the radiation source. For the radiation shielding purposes in the range of diagnostic imaging, the energy of K-absorption edges plays a key role. In this way, elements strongly absorb energy at points instantly above their particular edges. Considering the K-absorption edges of elements and the photoelectric effect, bilayers have been shown to offer significantly better attenuation per unit weight than lead-based composites, and essentially better attenuation per unit weight than even pure lead over a wide energy range up to mean photon energies of at least 66 keV[12].

The aim of this study was to introduce some lead free based materials as radiation attenuators, which have less density and toxicity in comparison with lead and assessment of their attenuation characteristics in the diagnostic radiology range (40, 60, 90 and 120 kVp). Finally, the radiation attenuation results of these materials are compared with lead and lead-based combinations. 

## Material and Methods

Many studies used Monte Carlo simulation (MCNP) to calculate photon attenuation and radiation dose[13-21]. It is proven that MCNP is valid enough for modeling photon transportation through materials and dose calculation[5, 15, 22-26]. In this study, MCNP4C was used to assess the attenuation effect of shielding materials.

MCNP is a computerized mathematical technique that is useful especially to solve complicated three-dimensional problems. This general-purpose code is based on the use of random numbers to investigate a statistical process such as the interaction of radiation with materials. Growing tendency to use Monte Carlo methods is due to the limitation of deterministic algorithms used by computer codes to calculate the precise answer of complex problems. MCNP is useful for photon, electron, neutron or coupled photon/electron/ neutron interaction with materials. In this code, energy ranges are used for neutron interaction with materials expanded from 10-11 MeV to 20 MeV, and the photon and electron energy ranges are from 1 keV to 1000 MeV. The input file created by the user is subsequently read by MCNP. This file comprises information about the materials specification, the characteristic of geometry and choice of cross-section assessments, the location and features of the photon, electron or neutron source, the kind of answers or tallies desired and any variance reduction methods used to increase efficiency. The above-mentioned areas should be defined as exactly as possible to get the most exact answer[27].

Elements were detected based on their k-edge density and nontoxicity characteristics. Different combinations of some elements (Cadmium (Cd), Tin (Sn), Barium (Ba), Tungsten (W), Lead (Pb)) were used  as shield and their density calculated. EPVC (Emulation poly vinyl chloride) and Si were used as matrix for different combinations. 

The simulation was performed in two situations: narrow and broad beam. In narrow beam situation, the beam of radiation was defined so narrowly and the attenuator materials were placed as far as possible from detector to reduce the arrival of scatter radiation to detector. This situation is called good geometry. If the scatter radiation can reach the detector, for example where attenuator material is placed near the detector or the beam is broad, the situation is called broad beam situation[28].

**Figure 1:**
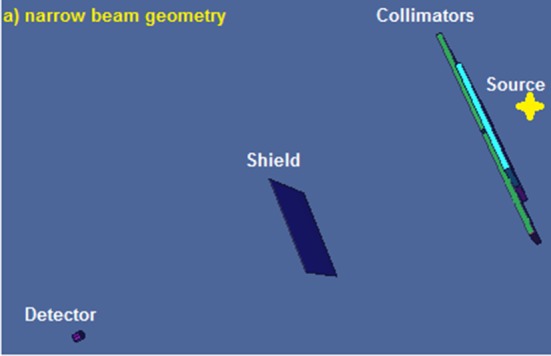
Narrow beam geometry defined in MCNP

In this study, narrow beam situation was a 60˚-angle cone source defined 50 cm away from the attenuator. The source was defined as cone beam to make the program more efficient and better geometry was achieved by 50 cm attenuator-detector distance[22] (Figure 1). This source was collimated with a lead collimator so that the field size on the attenuator was 5×5 cm2. The attenuator was a 10×10×0.1 cm3 plate of shield materials. Table 1 shows the composition and weight fraction of lead free materials used as shields in this study. In order to compare the attenuation characteristics of lead and lead free shield materials with lead shields, lead and two combinations of lead shields were simulated in the same geometries.

**Table 1:** Composition and weight fraction of lead and lead free materials used as shield.

**Material number**	**Material**	**Composition**	**Density (g/cm^3^)**
**1**	Pb	100%Pb	11.342
**2**	Pb-Si	67%Pb, 33%Si	4.98
**3**	Pb-EPVC	87%Pb, 13%EPVC	4.745
**4**	W-Si	67%W, 33%Si	5.70
**5**	W-Sn-Ba-EPVC	52.2%W, 30.45%Sn, 4.35%Ba, 13%EPVC	4.650
**6**	W-Sn-Cd-EPVC (1)	36.54%W, 46.11%Sn, 4.35%Cd, 13%EPVC	4.517
**7**	W-Sn-Cd-EPVC (2)	34.8%W, 43.5%Sn, 8.7%Cd, 13%EPVC	4.506

In narrow beam situation, a cylindrical lead shield is defined around the detector with a hole via 1 cm diameter above the detector and in front of the source. In this condition, the size of the hole would be small enough to remove the scatter radiations that reached the detector.

To assess the attenuation characteristics, the ratio of the intensity of transmitted radiation from the attenuator to the intensity of primary radiation (I/I^0^) was calculated. The simulation was run for 40, 60, 90 and 120 kVp with and without attenuator.

**Figure 2:**
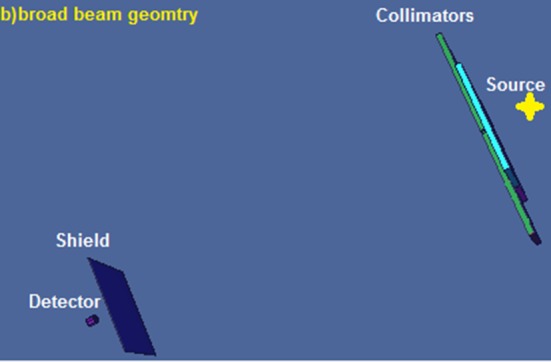
Broad beam geometry defined in MCNP

In broad beam geometry, the attenuator was placed in the immediate vicinity of the detector. In this situation, the lead shield around the detector was removed. Figure 2 illustrates the geometry of broad beam situation. The program of this geometry was run for 40, 60, 90 and 120 kVp with and without attenuator to calculate (I/I_0_).

The results of lead free shields were compared with lead-content attenuators in both situations. 

## Results

**Figure 3:**
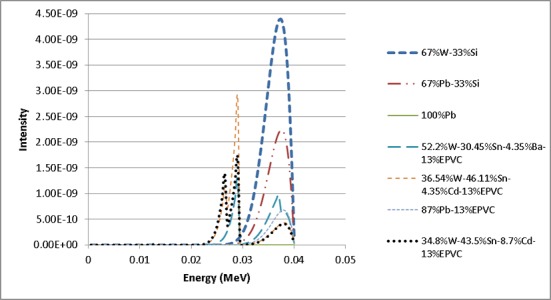
Energy spectrum of transmitted photons from attenuators at 40 kVp

**Figure 4:**
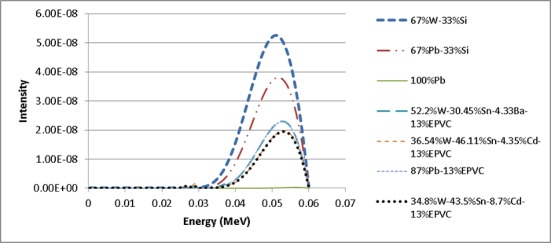
Energy spectrum of transmitted photons from attenuators at 60 kVp

**Figure 5:**
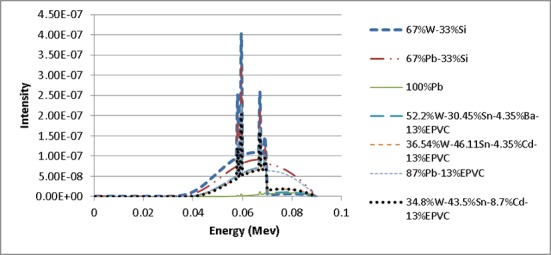
Energy spectrum of transmitted photons from attenuators at 90 kVp

**Figure 6:**
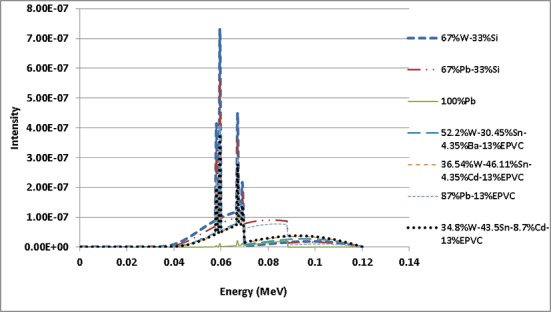
Energy spectrum of transmitted photons from attenuators at 120 kVp

The relative intensity (I/I^0^) of lead free and lead shielding materials in the narrow beam geometry at 40, 60, 90 and 120 kVp energies is shown in Table 2. Figures 3 to 6 represent the energy spectrum of the transmitted photon beams.

**Table 2:** The relative intensity of lead free and lead shielding materials in the narrow beam geometry at 40, 60, 90 and 120 kVp energies

**Materials**	** ρ (g/cm^3^) **	** I/I_0_**			
		40 kVp	60 kVp	90 kVp	120 kVp
Pb	11.342	3.02E-08	7.12E-05	6.34E-03	1.07E-02
Pb-Si	4.98	3.79E-04	1.75E-02	9.84E-02	1.39E-01
W-Si	5.70	7.96E-04	2.53E-02	8.66E-02	1.13E-01
Pb-EPVC	4.745	1.01E-04	9.69E-03	7.22E-02	1.04E-01
W-Sn-Ba-EPVC	4.650	1.99E-04	9.64E-03	5.10E-02	8.28E-02
W-Sn-Cd-EPVC (1)	4.517	2.46E-04	8.15E-03	5.00E-02	8.93E-02
W-Sn-Cd-EPVC (2)	4.506	2.01E-04	8.02E-03	5.01E-02	9.05E-02

The results of lead free and lead shielding materials in the Broad beam geometry at 40, 60, 90 and 120 kVp energies are shown in Table 3.

**Table 3:** The relative intensity of lead free and lead shielding materials in the broad beam geometry at 40, 60, 90 and 120 kVp energies

**Materials**	** ρ (g/cm^3^) **	** I/I_0_**			
		40 kVp	60 kVp	90 kVp	120 kVp
Pb	11.342	8.81E-10	1.07E-04	8.89E-03	1.64E-02
Pb-Si	4.98	4.82E-04	2.13E-02	1.14E-01	1.73E-01
W-Si	5.70	1.02E-03	3.08E-02	1.11E-01	1.54E-01
Pb-EPVC	4.745	1.28E-04	1.20E-02	8.48E-02	1.34E-01
W-Sn-Ba-EPVC	4.650	2.70E-04	1.20E-02	6.47E-02	1.07E-01
W-Sn-Cd-EPVC (1)	4.517	3.43E-04	1.04E-02	6.28E-02	1.12E-01
W-Sn-Cd-EPVC (2)	4.506	2.91E-04	1.03E-02	6.25E-02	1.12E-01

## Discussion

Pure lead was simulated just for comparison with other composites as a reference but not discussed in this part because it is not used in apron shields in pure form.

First, the results of the measurements in narrow beam situation will be discussed. According to Table 2, it seems that none of the lead free shields could be better than lead and lead-EPVC composites and it is probably because of the photoelectric effect, which is completely dominant in this range of energy. Pb–Si had a lesser metal fraction in comparison with material 4 to 7 so that this composition had bigger I/I^0^ than these materials.

In order to have a better comparison the percentage differences of material numbers 5 to 7, which are lead free shield materials, with material number 3 which is one of the most common lead shield material were calculated, is shown in Table 4. Percentage differences were calculated by the following equation:

$$\%Diff=\frac{I_{pb-EPVC}-I_{Materials\;(5-7)}}{I_{pb-EPVC}}\;(1)$$

**Table 4:** Percentage difference of materials 5-7 toward material 3 in narrow beam situation in 60, 90 and 120 kVp

**Material number**	**60 kVp**		**90 kVp**		**120 kVp**	
	I/I_0_	Diff%	I/I_0_	Diff%	I/I_0_	Diff%
3	9.69E-03	--------	7.22E-02	--------	1.04E-01	--------
5	9.64E-03	5.2	5.10E-02	29.4	8.28E-02	20.4
6	8.15E-03	15.9	5.00E-02	30.7	8.93E-02	14.1
7	8.02E-03	17.2	5.01E-02	30.6	9.05E-02	13.0

Using Eq 1, it is obtained that the bigger %Diff has better shielding performance.

According to Table 4, material numbers 6 and 7, in the ranges of 60, 90 and 120 kVp were found to provide lesser I/I^0^ than material numbers 2 and 3, as Figures 4 to 6 exhibit lower-transmitted x-ray intensity of these materials. In other words, two composites of (W-Sn-Cd-EPVC) showed a better attenuation in these ranges of energy.

Furthermore, for narrow beam, material number 5 showed worse radiation attenuation than material numbers 6 and 7 in 60 kVp, but almost the same attenuation in 90 kVp and even better attenuation in 120 kVp (Table 4).

Again, for broad beam situation, percentage differences of material numbers 5 to 7 with material number 3 were calculated (Table 5).

**Table 5:** Percentage difference of materials 5-7 toward material 3 in narrow beam situation in 60, 90 and 120 kVp

**Material number**	**60 kVp**		**90 kVp**		**120 kVp**	
	I/I_0_	Diff%	I/I_0_	Diff%	I/I_0_	Diff%
3	1.20E-02	--------	8.48E-02	--------	1.34E-01	--------
5	1.20E-02	0.00	6.47E-02	23.7	1.07E-01	20.1
6	1.04E-02	13.3	6.28E-02	25.9	1.12E-01	16.4
7	1.03E-02	14.2	6.25E-02	26.3	1.12E-01	16.4

According to tables 3 and 5, (W-Sn-Cd-EPVC) had the best performance in both broad and narrow beam situations. Our results showed that none of the considered lead free shields can be a preferential attenuator instead of lead in 40 kVp. This shows that lead is still effective and should be the material of choice for radiation protection in some energy ranges, as already has been stated with a modeling-based research[29]. However, material numbers 6 and 7 showed the best attenuation in 60 and 90 kVp but material number 5 presented the best attenuation performance in 120 kVp. These findings illustrated that the efficiency of the composite-type shields is more dependent on the photon beam energy than lead-only type shields as shown in previous research[8, 30]. This energy dependency of lead free shields demands the commitment of energy levels determination for all aprons. It is proved that in high energies in diagnostic radiology ranges, W-based shields in most cases as effective as lead-based shields[11]. Since any shields just weakly attenuate high-energy X-ray beams, utilizing thick layers of high-Z lead free materials like W-based shields, we can require significant protection while it is lighter and less toxic than lead-based shields.

## Conclusion

In this study, we have introduced four new lead free shielding materials three of which had lesser density but better attenuation performance in comparison with some lead-content composites like Pb-EPVC, in both situations of narrow and broad beam. The analysis of the data revealed that in low energies (40 kVp) lead and lead-content composites still were the best materials for radiation attenuation. However, in 60 and 90 kVp, (W-Sn-Cd-EPVC) composites showed better attenuation performance than lead and lead-content combinations. Composition of (W-Sn-Ba-EPVC) had the same results as (W-Sn-Cd-EPVC) composites in 90 kVp but better results in 120 kVp in both narrow and broad beam situations. Regarding the weight and toxicity of conventional lead-content shields, we think that these lead free shields can be considerable alternatives for lead in radiation shielding issue in a broad range of diagnostic radiology. Due to particular K-edge of each element, a single element cannot offer the best radiation protection for broad energy ranges. However, with suitable choice of elements for an especial range of energy, we can significantly improve shielding per unit weight over conventional lead-content shields. The present study has been investigated based on MCNP study. Therefore, further experimental study can support our findings. 
